# Association Between Cytometric Biomarkers, Clinical Phenotype, and Complications of Common Variable Immunodeficiency

**DOI:** 10.7759/cureus.52941

**Published:** 2024-01-25

**Authors:** Adam Markocsy, Anna Bobcakova, Otilia Petrovicova, Lenka Kapustova, Eva Malicherova Jurkova, Martina Schniederova, Jela Petriskova, Michal Cibulka, Michaela Hyblova, Milos Jesenak

**Affiliations:** 1 Department of Paediatrics, Martin University Hospital, Martin, SVK; 2 Department of Pulmonology and Phthisiology, Jessenius Faculty of Medicine, Comenius University in Bratislava, Martin, SVK; 3 Department of Paediatrics, Jessenius Faculty of Medicine, Comenius University in Bratislava, Martin, SVK; 4 Department of Clinical Immunology and Allergology, Martin University Hospital, Martin, SVK; 5 Department of Medical Genetics, Medirex a.s., Bratislava, SVK; 6 Department of Clinical Immunology and Allergology, Jessenius Faculty of Medicine, Comenius University in Bratislava, Martin, SVK

**Keywords:** transitional b cells, marginal zone b cells, b cell phenotypic profiling, immune dysregulation, common variable immunodeficiency

## Abstract

Background: Common variable immunodeficiency (CVID) is a heterogeneous group of immune disorders. The patients are classified according to the clinical manifestation with the infection-only phenotype (CVID_inf_) and CVID with immune dysregulation (CVID_id_).

Methods: We performed a retrospective clinical analysis of 64 CVID patients (34 males, 53.13%; mean age: 41.4 years; SD: ±21.4 years). We divided the patients into subgroups according to the clinical manifestation (CVID_inf_ and CVID_id_) and according to B cell phenotypic profiling after performing flow cytometry with the use of the EUROclass classification. We compared clinical manifestations, selected laboratory parameters, and therapy in these groups. All CVID_id_ patients were tested after the manifestation of complications associated with immune dysregulation and in eight patients during the immunosuppressive treatment (systemic corticosteroids and hydroxychloroquine).

Results: Two-thirds of patients in our cohort had symptoms resulting from immune dysregulation. Almost half of the patients had autoimmune complications. A higher proportion of marginal zone B cells was associated with autoimmune complications. A lower percentage of naïve B cells was connected to autoimmunity, whereas a lower proportion of transitional B cells was associated with rheumatic diseases and splenomegaly. Patients with lymphadenopathy had a higher percentage of double-negative T cells and a lower percentage of switched memory B cells. We performed molecular-genetic testing in 28% (n = 17) of patients and found a causal pathogenic variant in 23.5% (n = 4) of this group.

Conclusion: Based on our results, there is an association between specific cytometric parameters, clinical phenotype, and complications of CVID. The use of the subpopulations of B cells can be helpful in the diagnosis of these specific clinical complications in CVID patients and could help to personalise the therapeutic approach.

## Introduction

Common variable immunodeficiency (CVID) is a heterogeneous group of disorders that belong to predominantly antibody deficiencies with various clinical and immunological features such as increased susceptibility to infections, hypogammaglobulinemia, reduced switched memory B cell proportion, defective response to vaccination, autoimmune, granulomatous complications, or lymphoproliferative disorders (Table S1) [1]. There are many classification systems for CVID patients (Table S2). The patients can be classified according to the clinical manifestation of CVID with the infection-only phenotype (CVID_inf_) and CVID with immune dysregulation (CVID_id_) (autoimmune complications, lymphadenopathy with splenomegaly, granulomatous disease, hepatopathy, or enteropathy) [2].

There are four classification systems based on B-cell phenotypic profiling [3]. B cell development begins in the bone marrow from pluripotent hematopoietic stem cells through the pro-B cell (CD19-CD22+CD24-), pre-B cell (CD19+CD24++), and immature B cell (CD19+CD24++IgM+). We cannot see these cells in the peripheral circulation of healthy humans. Transitional B cells (CD19+CD21^norm^CD38+IgD-IgM+) leave the bone marrow for full maturation in secondary lymphoid organs (spleen, lymph nodes). Follicular (CD27-IgM+IgD+) and marginal zone B cells (CD19+CD27+IgM++IgD+) arise in the spleen and lymph nodes. Antigen recognition via the B cell antigen receptor (BCR) and the second signal (T cell-dependent or T cell-independent) is needed for B cell activation [4]. T cell-independent B cell response via innate receptor or extensive cross-linking of the BCR is characterised by extrafollicular plasma cell formation with marginal zone B cells [5]. Extrafollicular response forms plasma cells that can switch to IgG1 and are short-lived [6]. T cell-dependent B cell response via CD40-CD40L interaction is characterised by germinal centre formation with follicular B cells [7]. Germinal centres are crucial for class switch recombination, affinity maturation, selection, and expansion of antigen-specific B cell clones [8]. T-dependent activation is associated with the formation of naïve B cells (CD19+CD27-IgM+IgD+) and further memory B cells that undergo class switch recombination (switched memory B cells (CD19+CD27+IgM-IgD-)) or not (non-switched memory B cells (CD19+CD27+IgM+IgD-)). Post-germinal centre responses can create long-lasting humoral immunity [7]. EUROclass classification separates patients with nearly absent B cells (B-, <1%), severely reduced switched memory B cells (SmB-, <2%), and expansion of transitional (Tr^high^, >9%) or CD21^low^ B cells (CD21^low^, >10%). Reduction of switched memory B cells is associated with splenomegaly and granulomatous disease. Expansion of CD21^low^ B cells is associated with splenomegaly and expansion of transitional B cells with lymphadenopathy [9].

The molecular-genetic analysis supplies clinical diagnostic criteria. The 2022 European Society for Immunodeficiencies (ESID) genotype classification contains 23 genes associated with CVID [10]. We can determine the genetic diagnosis in 10-25% of CVID patients [11,12]. The well-known fact is that the deficiency of many other genes can cause CVID-like clinical and laboratory phenotypes, for example, genes associated with other groups of primary immunodeficiencies (combined immunodeficiencies with associated or syndromic features, diseases of immune dysregulation (BACH2 gene), autoinflammatory disorders (PLCG2 gene), and bone marrow failure (SAMD9, SAMD9L genes)) [13]. These findings support the importance of molecular-genetic analysis for determining the definitive diagnosis and treatment strategy [14].

It is important to find sensitive and specific diagnostic markers that can predict the clinical manifestation and complications of CVID patients. Early targeted management and therapy are associated with better prognosis and outcomes for patients. We aimed to analyse the differences in the selected laboratory parameters according to the clinical phenotype of CVID patients.

## Materials and methods

We enrolled CVID patients followed up in the Centre for Primary Immunodeficiencies at the University Hospital in Martin, Slovakia. Patients fulfilled the ESID diagnostic criteria for CVID (Table S1) after exclusion of all the secondary causes of hypogammaglobulinemia. All participants underwent a complete examination of immune status, evaluation of biochemical parameters, analysis of history data and clinical manifestations, and agreed to the study by signing the standard informed consent. The study and testing were performed after a long time from the first symptoms (1-25 years) because of diagnostic delay. The study was approved by the Ethical Committee “IRB00005636 Jessenius Faculty of Medicine, Comenius University in Martin IRB # 1” (EK 1/2022).

**Figure 1:**
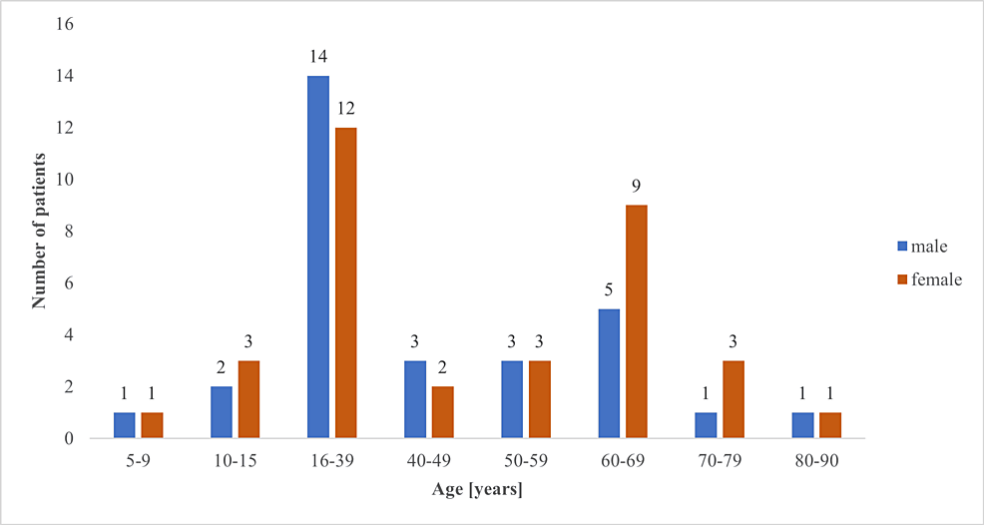
Age distribution of our CVID cohort. CVID: common variable immunodeficiency.

Our CVID cohort consisted of 64 patients (34 males, 30 females) with confirmed CVID diagnosis. The mean age of patients was 41.4 years (median ± SD: 37 ± 21.4 years). Patients aged from 16 to 39 years represented the largest group in our cohort (Figure 1).

We performed a retrospective analysis of the clinical manifestations and complications, laboratory findings, and treatment of the patients in our cohort. We assessed the presence of recurrent infections (respiratory, urinary tract), severe and invasive infections (pneumonia, meningitis, sepsis, severe herpetic infections), lymphadenopathy (confirmed by clinical, ultrasound, or CT examination), splenomegaly (proven by ultrasound examination), autoimmune complications (autoimmune cytopenia, celiac/sprue-like disease, autoimmune enteropathy, thyroiditis, arthritis, lupus-like disease), granulomatous complications (proven by CT examination and/or biopsy), and lymphoproliferative complications. In laboratory parameters, we evaluated the hypogammaglobulinaemia (IgG, IgA, and IgM values before immunoglobulin replacement therapy) and the response to specific vaccine antigens (diphtheric and tetanic toxoids, pneumococcal polysaccharides). In all patients, only one result of flow cytometry parameters was included in the study. It was the first possible result performed in our centre during the stable state of the disease course. All CVID_id_ patients were tested after the expression of complications associated with immune dysregulation and in eight patients during the immunosuppressive (IS)/immunomodulatory treatment (systemic corticosteroids, hydroxychloroquine). We performed the statistical analysis of flow cytometry parameters on the whole cohort first, then without patients taking immunosuppressive/immunomodulatory treatment (marked in text as value w/o IS).

The process of multiparametric flow cytometry was as follows: blood samples were collected into BD Vacutainer® tubes (Becton, Dickinson and Company, Franklin Lakes, NJ). For immunophenotyping, we used fluorescently labelled monoclonal antibodies in tube 1 (CD19-PC7, IgM-FITC, IgD-PE, CD27- PC5) and tube 2 (CD19-PC7, IgM-FITC, CD38-PC5, CD21-PE) (Beckman Coulter, Brea, CA) in an optimally titered volume. Briefly, 150 µl of whole blood was washed two times using phosphate-buffered saline (PBS). Cell sediment was suspended in 100 µl PBS and 50 microliter of cell suspension was incubated with monoclonal antibodies. After 30 minutes of incubation in the dark at room temperature, the erythrocytes were lysed with VersaLyse Lysing solution and then incubated for 10 minutes in the dark at laboratory temperature. Detection of B lymphocyte populations was executed using CytExpert software (Beckman Coulter, Brea, CA) and all analyses were performed on a DxFLEX flow cytometer (Beckman Coulter, Brea, CA).

**Figure 2:**
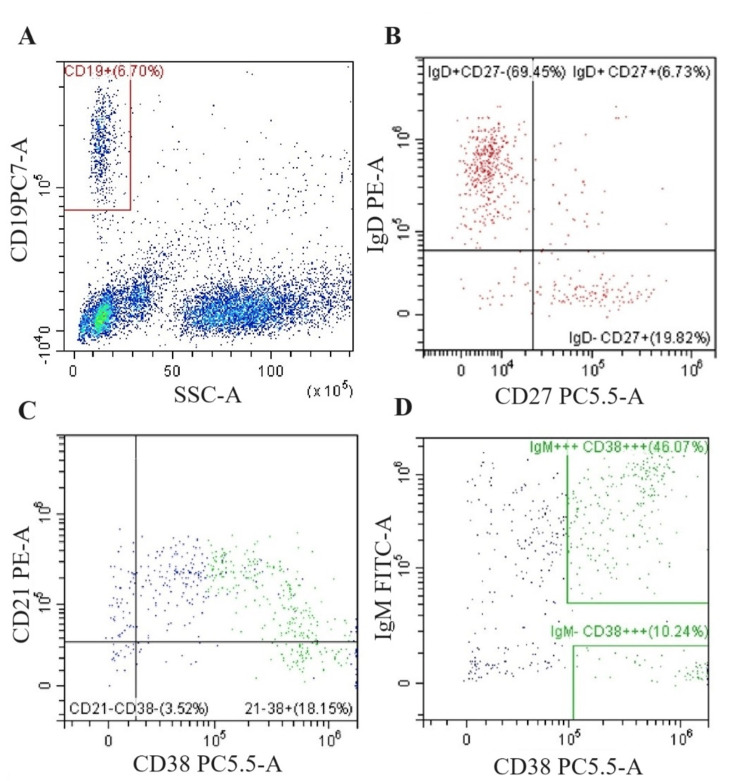
Hierarchical gating strategy used for immunophenotyping of B lymphocytes in patients with CVID. (A) Distribution based on side scatter analysis and expression of CD19. The population of all B lymphocytes expressing the CD19 marker is located in red square. (B) Distribution according to expression of IgD and CD27, naive CD19+IgD+CD27- (upper left quadrant), non-switched memory/marginal zone-like B lymphocytes CD19+IgD+CD27+ (upper right quadrant), and class-switched memory CD19+IgD-CD27+ (lower right quadrant). (C) Distribution according to the expression of CD38 and CD21. The population of activated B lymphocytes was determined as cells with low expression of CD21 and CD38 (lower left quadrant). (D) Distribution according to the expression of IgM and CD38, high expression of IgM and CD38 is characteristic for transitional B cells (upper green square), and lymphocytes expressing CD19 and CD38 with low expression of IgM were considered to be plasmablasts (lower green square). CVID: common variable immunodeficiency.

Peripheral mononuclear cells were defined by FSC (forward scatter) and SSC (side scatter). By manual gating, we defined the population of all B lymphocytes expressing the CD19 marker (Figure 2A).

Subsequently, we determined the population of different stages of B lymphocytes: naive CD19+IgD+CD27- (upper left quadrant), non-switched memory/marginal zone-like B lymphocytes CD19+IgD+CD27+ (upper right quadrant), and class-switched memory CD19+IgD-CD27+ (lower right quadrant) (Figure 2B). Based on the differential expression of IgM, CD38, and CD21 we determined three other B lymphocyte subsets. The population of activated B lymphocytes was determined as cells with low expression of CD21 and CD38 (lower left quadrant) (Figure 2C). High expression of IgM and CD38 is characteristic of transitional B cells (upper gate) and lymphocytes expressing CD19 and CD38 with low expression of IgM were considered to be plasmablasts (lower gate) (Figure 2D).

The absolute count of lymphocyte subsets (CD3+, CD19+, CD4+, CD8+, CD16+ CD56+ CD3-, CD4+/CD8+ ratio, CD3+/HLA-DR+) were determined. The relative number of peripheral B-lymphocyte subsets (marginal zone B cells (CD19+CD27+IgD+), naïve B cells (CD19+CD27-IgM+IgD+), switched memory B cells (CD19+CD27+IgM-IgD-), transitional B cells (CD19+CD27-CD21^norm^CD38++), CD21^low^ B cells (CD21^low^CD38^low^), plasma cells (CD19^low^CD21^norm^CD38+++IgM-(+)), and double-negative T cells (CD3+CD4-CD8-TCRα/β) were determined.

We evaluated the treatment: immunoglobulin substitution therapy-conventional subcutaneous, facilitated subcutaneous, and intravenous application, and antibiotic prophylaxis (co-trimoxazole, azithromycin, valaciclovir). Eight CVID_id_ patients (21.1%) were tested during the immunosuppressive/immunomodulatory treatment. Four patients on therapy with systemic corticosteroids, three patients on treatment with hydroxychloroquine, and one patient with both. Systemic corticoids (prednisone) were used in small doses (0.01-0.3 mg/kg).

The patients were divided into sub-groups according to the clinical manifestation in CVID_inf_ (patients with infections only) and CVID_id_ (patients with at least one of the following: autoimmune complications, lymphadenopathy with splenomegaly, granulomatous disease, hepatopathy, and enteropathy), and according to B cell phenotypic profiling after performing flow cytometry with use of EUROclass. We compared clinical manifestations, laboratory findings, and therapy in these groups.

For statistical analysis, we use Jamovi computer software. For nominal data, we use the χ² test. For continuous data, we established mean, median, standard deviation (SD), standard error of the mean (SE), and 95% confidence interval (95% CI) for mean lower- and upper-bound. Continuous data are tested for normal distribution with the use of the normality test (Shapiro-Wilk) and QQ plot. Statistical differences are determined by parametric independent samples Student's t-test or non-parametric Mann-Whitney U test. For comparison of more than two groups, statistical differences are determined by parametric one-way ANOVA or by non-parametric Kruskal-Wallis testing using Dwass-Steel-Critchlow-Fligner (DSCF) pairwise comparison analysis. Differences are considered statistically significant at p < 0.05.

We performed molecular-genetic testing on 17 patients who were available and agreed for genetic examination. For testing, we used isolated peripheral blood mononuclear cells (PBMCs) derived from the patient's blood. We used massive parallel sequencing focused on the panel for primary immunodeficiencies (Invitae Primary Immunodeficiency Panel, Invitae Corporation, CLIP Laboratory Centre, Prague, Czech Republic), 342 genes virtual panel for primary immunodeficiencies with the use of clinical exome (Illumina NextSeq, SOPHiA Clinical Exome Solution v3 kit (Sophia Genetics, Saint-Sulpice, Switzerland), Sophia DDM analysis, Medirex a.s., Bratislava, Slovakia), or whole exome sequencing (Illumina NextSeq, Twist Human Core Exome Kit, Ingenuity Variant Analysis (Qiagen, Hilden, Germany), Medirex a.s., Bratislava, Slovakia). We studied the genotype-phenotype correlations in patients with confirmed pathogenic or likely pathogenic variants in genes associated with primary immunodeficiencies.

## Results

In our CVID cohort, two-isotype hypogammaglobulinaemia (IgG, IgA) was detected in 34.4% (n = 22) of patients and three-isotype hypogammaglobulinaemia (IgG, IgA, IgM) was detected in 65.6% (n = 42) of patients. We found a significantly higher CD4/CD8 ratio in patients with two-isotype hypogammaglobulinaemia in comparison with three-isotype hypogammaglobulinaemia (p = 0.003). The facilitated subcutaneous immunoglobulin (fSCIG) replacement therapy was used in 87.5% (n = 56) of patients, intravenous immunoglobulin (IVIG) replacement therapy in 4.7% (n = 3), and 7.8% (n = 5) did not agree with the substitution therapy and refused it. Antibiotic prophylaxis due to frequent or complicated infection complications, despite the optimised immunoglobulin replacement therapy, was indicated in 17.2% (n = 11). The demographic and clinical characteristics of the cohort are summarised in Table 1.

**Table 1:** Demographic, laboratory, and clinical characteristics of the analysed cohort of CVID patients. CVID: common variable immunodeficiency; LAP: lymphadenopathy; fSCIG: facilitated subcutaneous immunoglobulin.

Parameter	Number (percentage)		
	Whole cohort	CVID_inf_	CVID_id_
Number of patients	64 (100%)	26 (40.6%)	38 (59.4%)
Males	34 (53.1%)	16 (61.5%)	14 (36.8%)
Females	30 (46.9%)	10 (38.5%)	24 (63.2%)
Infectious complications	56 (88%)	23 (88.5%)	33 (86.8%)
Severe infections	22 (34%)	10 (38.5%)	12 (31.6%)
Allergy	27 (42.2%)	9 (34.6%)	18 (47.4%)
Autoimmune complications	27 (42%)	0	25 (65.8%)
Lymphoproliferative disease	5 (8%)	2 (7.7%)	3 (7.9%)
Lymphadenopathy (LAP)	22 (34%)	5 (19.2%)	17 (44.7%)
Splenomegaly	13 (20%)	4 (15.4%)	9 (23.7%)
LAP + splenomegaly	8 (12.5%)	0	8 (21%)
Granulomatous complications	11 (17%)	0	11 (28.9%)
fSCIG	56 (87.5%)	24 (92.3%)	32 (84.2%)
IVIG	3 (4.7%)	0	3 (7.9%)
Without Ig substitution	5 (7.8%)	2 (7.7%)	3 (7.9%)
Antibiotic prophylaxis	11 (17.2%	5 (19.2%)	6 (15.8%)
Immunosuppressive treatment (prednisone)	5 (8%)	0	5 (13.2%)
Immunomodulation treatment (hydroxychloroquine)	4 (6.3%)	0	4 (10.5%)
Two-isotype hypogammaglobulinaemia	22 (34.4%)	10 (38.5%)	12 (31.6%)
Three-isotype hypogammaglobulinaemia	42 (65.6%)	16 (61.5%)	26 (68.4%)
Diagnostic vaccination (pneumococcal) (no response/performed)	16/29 (55.2%)	9/12 (75%)	8/17 (47%)
Diagnostic vaccination (diphtheria) (no response/performed)	22/29 (75.9%)	7/12 (58.3%)	15/17 (88.2%)
Diagnostic vaccination (tetanus) (no response/performed)	20/29 (69%)	7/12 (58.3%)	13/17 (76.5%)

**Figure 3:**
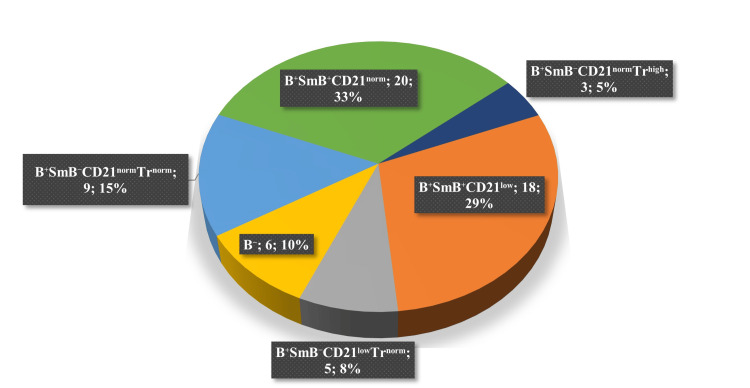
Division of the patients according to the EUROclass classification. The distribution of patients according to the EUROclass classification after the examination of B cell phenotyping by flow cytometry. The EUROclass classification separates patients according to the proportion of B cells, switched memory B cells, transitional B cells, and CD21^low^ B cells - nearly absent B cells (B-, <1%), the proportion > 1% B cells (B+), severely reduced switched memory B cells (SmB-, <2%), the proportion > 2% of SmB (SmB+), the expansion of transitional B cells (Tr^high^, >9%), without the expansion of transitional B cells (Tr^norm^, <9%), and the expansion of CD21^low^ B cells (CD21^low^, >10%), without the expansion of CD21^low^ B cells (CD21^norm^, <10%) [9]. Group B+SmB+CD21^norm^ had 20 patients (32.8%), group B+SmB+CD21^low^ had 18 patients (29.5%), group B⁺SmB⁻CD21^norm^Tr^norm^ was observed in nine patients (18%), group B⁻ had six patients (10%), group B⁺SmB⁻CD21ˡᵒʷTr^norm^ had five patients (8%), and group B⁺SmB⁻CD21^norm^Trʰⁱᵍʰ was observed in three patients (5%). We did not identify any patient from class B+SmB-CD21ˡᵒʷTrʰⁱᵍʰ.

We analysed the distribution of the patients according to the EUROclass classification. The group B+SmB+CD21^norm^ with 20 patients (32.8%) was the most frequent, and the second most frequent group was B+SmB+CD21^low^ with 18 patients (29.5%). We did not identify any patient from class B+SmB-CD21^low^Tr^high^ (Figure 3).

Three patients did not undergo the examination of B cell phenotyping by flow cytometry because they refused the testing. Therefore, they were excluded from this analysis. We did not find statistically significant differences in clinical manifestation between these groups.

**Figure 4:**
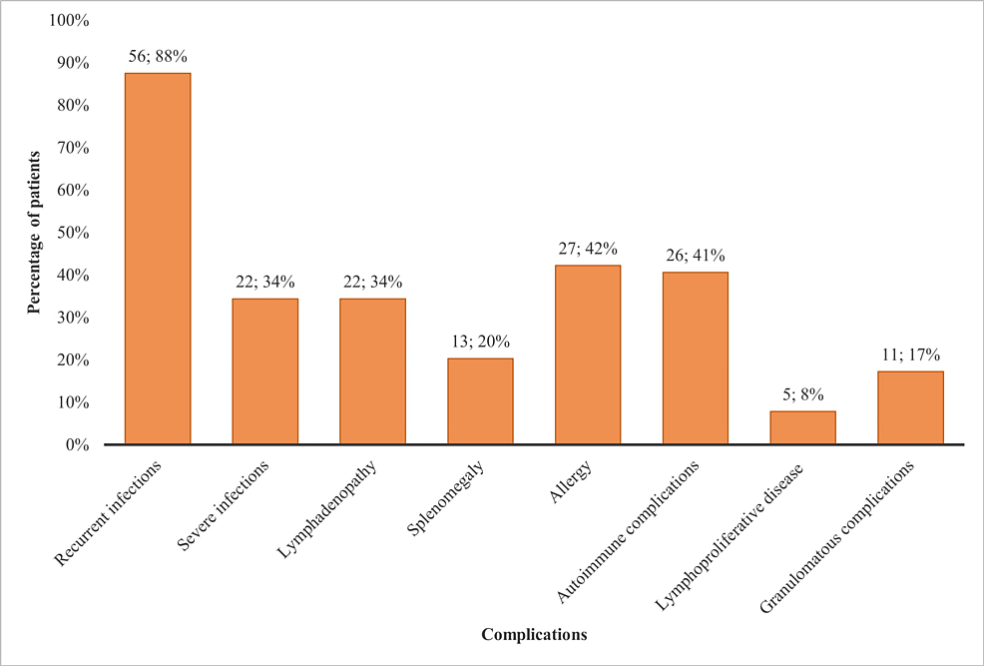
Complications of CVID patients. The first number above each column represents the number of patients with specific complications, and the second number represents the percentage of the cohort total. CVID: common variable immunodeficiency.

Infectious complications were present in 88% (n = 56) of patients, with severe and invasive infections in 34% (n = 22). Two patients developed bronchiectasis. The lymphoproliferative disease was found in 8% (n = 5), lymphadenopathy in 34% (n = 22), splenomegaly in 20% (n = 13), and granulomatous complications in 17% (n = 11) of our patients (Figure 4). Of patients with granulomatous complications, nine patients had granulomatous lymphocytic interstitial lung disease (GLILD), and two patients had sarcoidosis/sarcoid-like disease.

**Figure 5:**
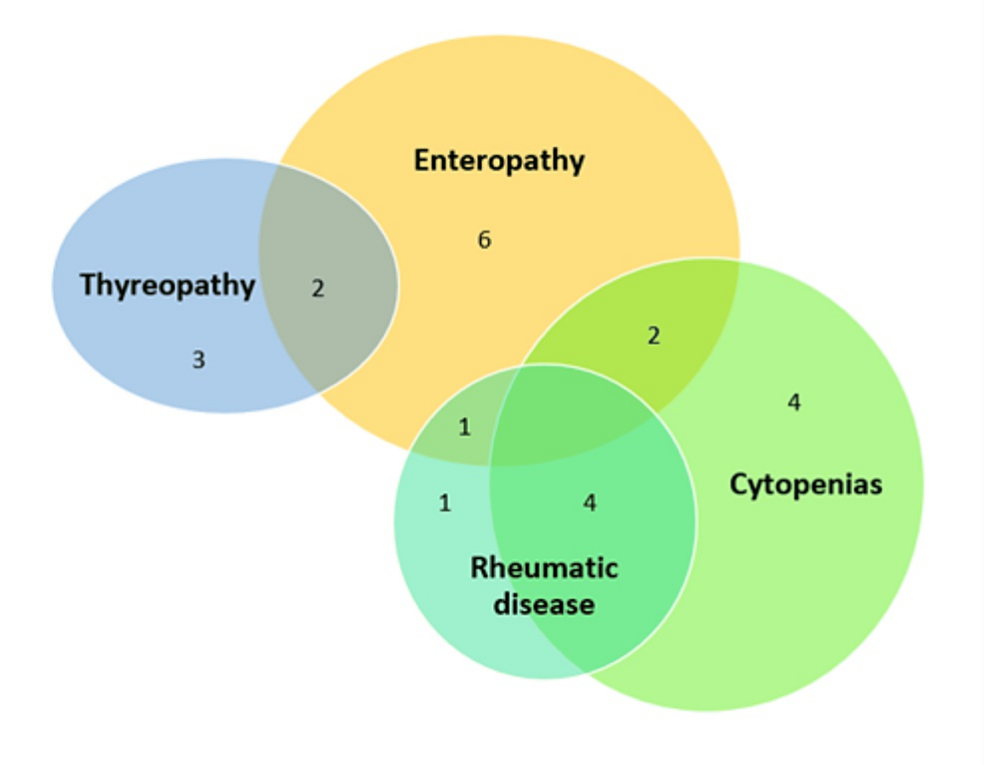
Venn diagram of autoimmune complications. Numbers in areas of the Venn diagram indicate the number of patients with a specific combination of complications.

Autoimmune complications were present in 42% (n = 27). Of these patients, 40.7% (n = 11) had enteropathy, 37% (n = 10) had cytopenias, 22.2% (n = 6) had rheumatic disease, and 18.5% (n = 5) had autoimmune thyroiditis. There is a Venn diagram of autoimmune complications in Figure 5.

We analysed our cohort according to the clinical manifestations. A total of 26 patients (40.6%) had CVID_inf_ and 38 patients (59.4%) had CVID_id_. In our cohort, we found a borderline statistical difference in the development of CVID_id_ according to gender (p = 0.052). Women seemed more likely to develop CVID_id_ than men.

**Figure 6:**
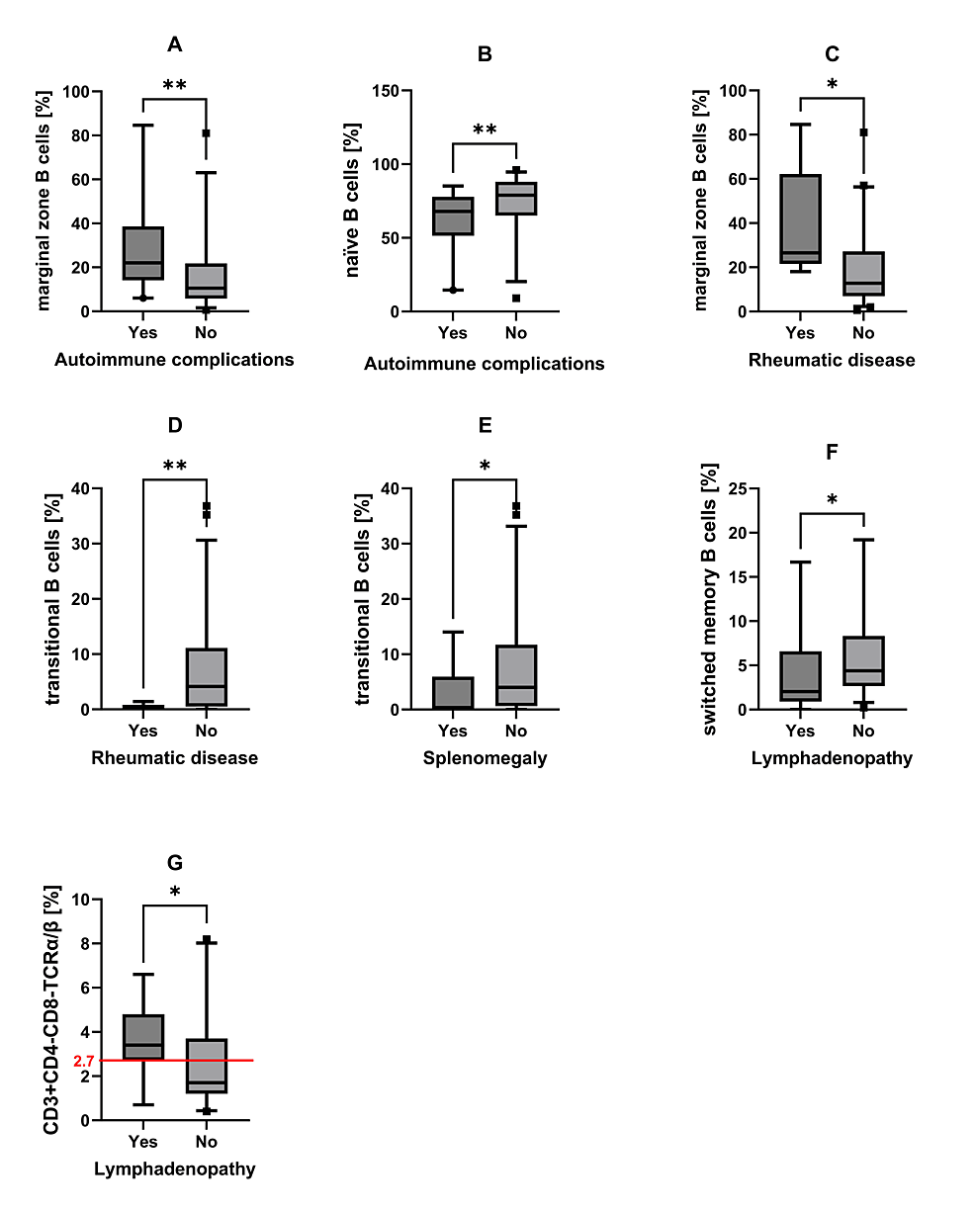
Proportion of selected flow cytometry parameters according to the presence of complications. Groups of CVID patients with (Yes) and without (No) specific complications are compared according to the following: (A) the percentage of marginal zone B cells (autoimmune complications, p = 0.001); (B) the proportion of naïve B cells (autoimmune complications, p = 0.01); (C) the proportion of marginal zone B cells (rheumatic disease, p = 0.013); (D) transitional B cells (rheumatic disease, p = 0.007); (E) transitional B cells (splenomegaly, p = 0.046); (F) switched memory B cells (lymphadenopathy, p = 0.034); and (G) the percentage of CD3+CD4-CD8-TCRα/β (lymphadenopathy, p = 0.029). Mann-Whitney U test was used in graphs A-F. χ² test was used in graph G (comparison of the group of patients with >2.7% of double-negative T cells (DNT) cells in peripheral blood to patients with <2.7%). If a p-value is less than 0.05, it is flagged with one star (*). If a p-value is less than 0.01, it is flagged with two stars (**). If a p-value is less than 0.001, it is flagged with three stars (***). CVID: common variable immunodeficiency.

CVID patients with autoimmune complications had a statistically significantly higher percentage of marginal zone B cells (p = 0.001, Figure 6A, value w/o IS: p = 0.007) and a lower proportion of naïve B cells (p = 0.01, Figure 6B¸ value w/o IS: p = 0.012).

In CVID patients with rheumatic disease, we also found a higher proportion of marginal zone B cells (p = 0.013, Figure 6C, value w/o IS: p = 0.049) and a lower percentage of transitional B cells (p = 0.007, Figure 6D, value w/o IS: p = 0.159). Patients with splenomegaly had a lower percentage of transitional B cells (p = 0.046, Figure 6E, value w/o IS: p = 0.026). We found a statistically significantly higher occurrence of lymphadenopathy in CVID patients with a lower percentage of switched memory B cells (p = 0.034, Figure 6F, value w/o IS: p = 0.056) and a higher percentage of double-negative T cells (above 2.7% of B cells in peripheral blood; p = 0.029, Figure 6G, value w/o IS: p = 0.045). Patients with granulomatous disease had a higher percentage of natural killer (NK) cells (p = 0.027, value w/o IS: p = 0.038). Patients without the granulomatous disease had a higher absolute number (p = 0.025, value w/o IS: p = 0.014) and percentage of B cells (p = 0.032, value w/o IS: p = 0.015). Patients with allergy had higher absolute number (p = 0.027, value w/o IS: p = 0.062) and percentage (p = 0.048, value w/o IS: p = 0.113) of CD4 T cells, and CD4/CD8 ratio (p = 0.006, value w/o IS: p = 0.017). We did not find statistically significant differences in parameters of flow cytometric immunophenotyping comparing the subgroup of CVID_inf_ and the separate subgroups of CVID_id_ patients (autoimmune complications, lymphadenopathy with splenomegaly, granulomatous disease, hepatopathy, and enteropathy).

We also studied clinical manifestation according to IgM values. We did not find a statistically significant difference in patients with CVID_id_ compared to the CVID_inf_ group (p = 0.763, value w/o IS: p = 0.879), patients with and without the granulomatous disease (p = 0.120, value w/o IS: p = 0.134), and patients with and without splenomegaly (p = 0.107, value w/o IS: p = 0.064).

By studying complications of CVID patients with SmB- (n = 17), we found that 53% (n = 9) had lymphadenopathy and 47% (n = 8) had autoimmune complications. We found statistically insignificant differences for autoimmune complications (p = 0.365), splenomegaly (p = 0.770), and granulomatous disease (p = 0.308) between the SmB- and SmB+ groups. Patients with CD21^low^ (n = 22) phenotype developed autoimmune complications in 45% (n = 10) and lymphadenopathy in 41% (n = 9). In comparison with the CD21^norm^ phenotype (n = 33), there were statistically insignificant differences for these two parameters (p = 0.365 and p = 0.418, respectively) and for splenomegaly (p = 0.074).

We performed molecular-genetic testing in 28% (n = 17) of the patients with CVID laboratory and clinical phenotype and found a causal pathogenic variant in 23.5% (n = 4) of this group. We found one pathogenic variant in the NFKB1 gene in a father and his daughter, one pathogenic variant in the TNFRSF13B gene, and one pathogenic variant in the MAGT1 gene associated with X-linked immunodeficiency with magnesium defect, Epstein-Barr virus infection, and neoplasia (XMEN syndrome).

## Discussion

CVID is the most common clinically significant primary immunodeficiency in clinical practice. It is associated with a broad spectrum of infectious and non-infectious (usually autoimmunity and/or lymphoproliferation) complications [15]. In our cohort of CVID patients, we showed that a higher proportion of marginal zone B cells in blood is associated with autoimmune complications and rheumatic disease. A lower percentage of naïve B cells is connected to autoimmunity. A lower proportion of transitional B cells is associated with rheumatic disease and splenomegaly. Patients with lymphadenopathy had a higher percentage of double-negative T (DNT) cells and a lower percentage of switched memory B cells.

Recurrent infections are a common sign of CVID patients but do not need to dominate the clinical picture. Autoimmunity is one of the most frequent non-infectious complications, seen in 25-30% of CVID patients [16]. In our study, autoimmune complications are even more prevalent (42% of patients). Two-thirds of patients have symptoms resulting from immune dysregulation. Enteropathy and cytopenias are the most common complications. There is a strong association between the presence of cytopenias and rheumatic disease. In our cohort, women seem more likely to develop CVID_id_ than men. This observation is in accordance with the knowledge that women are more likely to develop autoimmune disorders than men [17]. The treatment of patients with immune dysregulation is more complicated and they usually need specific management.

CD21 is a co-receptor to the BCR. Simultaneous triggering of the BCR and CD21 lowers the threshold for B cell activation [18]. Expression of CD21 is important for B cell tolerance and regulatory functions [19]. CD21^low^ B-cells contain mostly autoreactive unresponsive clones [20]. Expansion of CD21^low^ B cells is associated with chronic immune stimulation or autoimmune diseases (systemic lupus erythematosus, rheumatoid arthritis). CD21^low^ B cells are considered to serve as antigen-presenting cells (APCs) [21]. According to the literature, expansion of CD21^low^ B cells is associated with splenomegaly, expansion of transitional B cells with lymphadenopathy, and reduction of switched memory B cells is associated with splenomegaly and granulomatous disease [9]. In our cohort, we did not confirm this observation probably due to a small number of studied patients.

Marginal zone B cells (MBZ) are situated in splenic follicles and lymph nodes where they are responsible for an initial, rapid defence reaction against blood-borne pathogens by production of IgM. MBZ express polyreactive BCRs and low-affinity antibodies. The expansion of MBZ and simultaneous stimulation with pathogen- or damage-associated molecular patterns (PAMPs/DAMPs) and self-antigen is related to presentation of self-antigen, activation of self-reactive CD4+ T helper cells, secretion of autoantibodies, and development of autoimmunity [22,23]. A higher proportion of MBZ in our cohort was associated with autoimmune complications and rheumatic disease (p = 0.001, value w/o IS: p = 0.007 and p = 0.013, value w/o IS: p = 0.049). A lower percentage of naïve B cells was also connected to autoimmunity (p = 0.01, value w/o IS: p = 0.012).

Transitional B (TrB) cells represent a developmental stage between immature B cells in the bone marrow and mature peripheral B cells. TrB cells are one of the regulatory B cell subpopulations due to the production of IL-10 and regulation of effector CD4+ T helper cells maturation and conversion to regulatory T cells (Treg). Indeed, TrB cells suppress CD8+ T cell responses, the proliferation of autoreactive CD4+ T cells, and the differentiation of CD4+ T cells into Th1 and Th17 cells that decreases the production of pro-inflammatory cytokines (tumour necrosis factor-alpha, interferon-gamma (IFN-γ), and IL-17) [24,25]. A lower proportion of TrB in our cohort was associated with rheumatic disease and splenomegaly (p = 0.007, value w/o IS: p = 0.159 and p = 0.046, value w/o IS: p = 0.026). Azizi et al. showed differences in naïve, marginal zone, and transitional B cells in CVID patients with and without autoimmune complications, although these differences were not significant [26]. In our cohort, we show significant differences in these parameters.

DNT cells are a subset of mature T lymphocytes, which have intrathymic or extrathymic (downregulation of CD4/CD8 expression) origin. They are observed in peripheral blood, secondary lymphoid organs, and various tissues. DNT cells are expanded in patients with autoimmune lymphoproliferative syndrome, inflammatory autoimmune conditions (systemic lupus erythematosus, Sjögren's syndrome, psoriasis) where DNT cells infiltrate target organs and produce pro-inflammatory cytokines (IL-17, IFN-γ) [27-29]. In our cohort, CVID patients with lymphadenopathy had a higher percentage of DNT cells (p = 0.029, value w/o IS: p = 0.045).

In our study, we performed a complex and detailed examination of complications and detailed examination of immune profiles. The limitations of our study are the small sample of patients, the margin of error possibility, diagnostic delay, different disease courses with developed complications at the time of testing, only a small part of the cohort underwent genetic testing, and data were compared only between the groups of CVID patients without healthy controls.

## Conclusions

The prognosis of CVID patients should not be based only on one solo marker. A combination of biomarkers would be more appropriate and precise. Clinical diagnostic criteria for CVID and classification systems are still not sufficient to predict the clinical manifestation and complications. Genetic testing could be helpful in this issue. One patient with XMEN syndrome was excluded from the group of CVID patients and got targeted specific management. We assume that there will be more patients with other genetic diagnoses in our cohort that will require genetic testing, as well as in the rest of our studied patients.

CVID is a complex disease that requires a multidisciplinary approach. Selected flow cytometric parameters can be used for the prediction of clinical complications. The use of the combination of the selected laboratory and clinical biomarkers would be the best way to adapt and personalize the management of these patients.

## Appendices

Table S1

**Table 2:** Clinical criteria for a probable diagnosis of CVID according to the ESID. Modified according to Seidel et al. (2019) [1]. CVID: common variable immunodeficiency; ESID: European Society for Immunodeficiencies.

At least one of the following:	And at least one of the following:	And:	Alternative diagnosis
Increased susceptibility to infection	Poor antibody response to vaccines	Marked decrease of IgG and IgA with or without low IgM levels	Patients < 4 years old
Autoimmune manifestation			
Granulomatous disease	Low-switched memory B cells	Secondary causes of hypogammaglobulinaemia have been excluded	Patients with evidence of profound T-cell deficiency
Unexplained polyclonal lymphoproliferation			
Affected family member with antibody deficiency			

Table S2

**Table 3:** CVID classification system. Modified according to Berbers et al. (2021) [2], Yazdani et al. (2017) [3], and Tangye et al. (2022) [10]. CVID: common variable immunodeficiency; ↓: reduction; SmB: switched memory B cells; Tr: transitional B cells; MZB: marginal zone B cells; LOF: loss of function.

Classifications		
Clinical manifestation	B cell phenotypic profiling	Genotype classification
1. Infection phenotype (CVID_inf_)	1. Freiburg classification	Common variable immune deficiency with no gene defect specified
Infections only	I. SmB⁺ < 0.4%	Activated p110δ syndrome (APDS)
2. Immune dysregulation phenotype (CVID_id_)	I.a CD21^low^ B-cells > 20%	PTEN deficiency (LOF)
At least one of the following:	I.b CD21^low^ B-cells < 20%	CD19 deficiency
Autoimmune complications	II. SMB⁺ > 0.4%	CD81 deficiency
Granulomatous disease	2. Paris classification	CD20 deficiency
Hepatopathy	MB0. CD27⁺ B-cells < 11%	CD21 deficiency
Enteropathy	MB1. SMB < 8%, CD27⁺ B-cell > 11%	TACI deficiency
Lymphadenopathy with splenomegaly	MB2. Others	BAFF receptor deficiency
	3. EuroCLASS	TWEAK deficiency
	B^⁺^SmB⁻CD21ˡᵒʷTrʰⁱᵍʰ	TRNT1 deficiency
	B⁺SmB⁺CD21ˡᵒʷ	NFKB1 deficiency
	B⁺SmB⁻CD21ˡᵒʷTrⁿᵒʳᵐ	NFKB2 deficiency
	B⁻	IKAROS deficiency
	B⁺SmB⁻CD21ⁿᵒʳᵐTrⁿᵒʳᵐ	IRF2BP2 deficiency
	B⁺SmB⁺CD21ⁿᵒʳᵐ	ATP6AP1 deficiency
	B⁺SmB⁻CD21ⁿᵒʳᵐTrʰⁱᵍʰ	ARHGEF1 deficiency
	4. B-cell patterns classification	SH3KBP1 (CIN85) deficiency
	1. ↓ Tr and SmB	SEC61A1 deficiency
	2. Normal Tr and ↓ of naive mature, MBZ and SmB	RAC2 deficiency
		Mannosyl-oligosaccharide glucosidase deficiency
	3. ↓ MBZ and SmB	PIK3CG deficiency
	4. Isolated ↓ SmB	BOB1 deficiency
	5. Normal MBZ and SmB	Other genes associated with primary immunodeficiencies
